# Get online to support wellbeing of graduate students

**DOI:** 10.7554/eLife.53178

**Published:** 2019-11-13

**Authors:** Liesl A Krause, Susanna L Harris

**Affiliations:** 1 Department of Technology Leadership and InnovationPurdue University Polytechnic InstituteWest LafayetteUnited States; 2 Department of Microbiology and ImmunologyUniversity of North Carolina at Chapel HillChapel HillUnited States

**Keywords:** mental health, academia, graduate students, online resources

## Abstract

Universities should use online resources to help graduate students who are struggling with their mental health to access appropriate support.

Mental health issues are a serious problem in academia, especially among graduate students where around 25–40% face mental health concerns (Evans et al., 2018; Levecque et al., 2017; Barreira et al., 2018). Moreover, about half of those students will likely not receive treatment (SAMHSA, 2019). These figures highlight the pressing need for the academic community to both offer more support for graduate students struggling with their mental health and remove the barriers that prevent those students from getting help where it already exists.

We are two PhD students at universities in the United States. We both also volunteer with PhD Balance, an online community dedicated to sharing resources and stories to empower graduate students professionally and personally. Susanna founded PhD Balance in 2018 (originally called "The PhDepression") to find other students managing mental illness while in graduate school. Liesl joined after her own mental health experiences lead her to reach out for the resources and support provided by groups like PhD Balance. At the time of writing, the PhD Balance community includes over 36,000 followers, across Twitter and Instagram, from six continents, and these numbers continue to grow.

In this article, we highlight factors that may prevent graduate students from accessing mental health support as it exists now, before exploring how online tools can overcome barriers to access with examples drawn from personal experiences within the education system in the United States. Finally, we recommend concrete steps that institutions can take to better support the mental wellbeing of graduate students via online resources.

## Existing support options

The factors affecting the mental health of graduate students are complex, and each student’s experience is unique. Yet, based on conversations with thousands of graduate trainees and other academics through PhD Balance, we have identified some common themes that are often reported.

The transition from undergraduate to graduate studies can lead many students to shift how they view the world and how they perceive themselves in it (Dunn et al., 2008). This potentially jarring and stressful experience can lead students to question their aptitude for the field. This, in turn, may reduce their likelihood of acknowledging signs or symptoms of mental health distress and seeking help (Parkman, 2016). Additionally, graduate students often feel guided, either explicitly or implicitly, to emulate their mentors (Dunn et al., 2008). If a student is told to get rest or spend time with friends but sees their mentor often working late and seemingly neglecting other responsibilities, the student is more likely to mirror the behavior than take the advice. Further, PhD advisors may also repeat the behavior of others, potentially leading to academic "hazing". This occurs when a mentor or superior challenges a new student to essentially 'prove themselves worthy' because that is how they were treated when they entered the field (Dominguez and Hager, 2013), even though the pressure can be harmful to the mental wellbeing and productivity of the student. Lastly, through experience we know that graduate school can be isolating for a number of reasons. When a student is struggling, they may not know how to get help, and if a student feels isolated and alone, it only serves to make the struggle worse.

Though not all students struggling with their mental health will seek help from their university, institutions of higher education have reported a nearly 30% increase in the use of university mental health resources between 2010 and 2015 (Winerman, 2017). Some institutions, for example, offer courses on time-management and work/life balance, or even classes in meditation or yoga; however, such activities appear to, at best, offer short-term benefits and are often ill-attended by graduate students (Barbosa et al., 2013; Dunn et al., 2008).

Most universities list their in-person resources on their websites, allowing students to access some basic information without directly contacting a university employee. However, there is a lack of data around which universities supplement these offerings through the curation of more detailed web pages or access to external online resources. One example of an online resource being leveraged by a university is the "WellTrack" web app at Purdue University, which allows students to track and monitor their own mental health and provides coping and mitigation methods to the students separate from the university’s counseling program. It would be useful to know whether online interventions are being implemented more widely and how effective they are in supporting both undergraduate and graduate students.

One common issue we have encountered with mental health interventions at universities is that they are almost always tailored to undergraduates, because undergraduates typically make up the bulk of the student population on any campus. However, factors such as housing, finances and stage of life will vary greatly between these two groups (Hunt and Eisenberg, 2010). The stories collected through PhD Balance show that a graduate student’s experiences are often unlike those of undergraduates studying at the same university but share similarities with PhD students across institutions and topics of study.

We believe that universities need to shift the focus of at least some of their mental health provision to better serve graduate students. We are aware that offering more nuanced resources may pose a major challenge for university administrations facing budgetary and regulatory restrictions. However, while they cannot be replacements for in-person interventions, we believe that online resources may be a convenient way for graduate students to seek help that is more tailored to their unique needs.

## Benefits of online resources

Timely response to a mental health crisis can be the difference between life and death. Students may feel uncomfortable contacting emergency medical services, such as calling 911 in the United States. They may also not be financially capable of using these emergency services if there is a charge, because money concerns are common problems faced by graduate students (Sowell et al., 2008). Around-the-clock access to responsive providers may be critical in supporting students outside of regular academic hours. Suicide rates peak in the spring regardless of hemisphere (Cho and Lee, 2018), which is a transition time for universities as they prepare for the end of the academic year – meaning that students may unfortunately also experience lapses in access to student health services at this time. One solution to partially address these concerns is for institutions to clearly list crisis hotlines, both local and national, and other free resources prominently on their websites.

Thoughtful use of websites and digital media could also help in guiding users to relevant resources, whether housed online or available in person or via phone. Online resources may complement existing systems; for example, if a student is concerned about their mental health, they can use the online version of the Patient Health Questionnaire (i.e., PHQ-9) to assess their indicators for anxiety and depression. While the results of this evaluation would need to be verified by a certified mental health provider, the student may feel more comfortable with and capable of reaching out to university mental care facilities when they are already equipped with this information.

Though there are a number of movements to "end the stigma", negative perspectives of mental illness are pervasive in academia (Mannarini and Rossi, 2018). The fear of facing such stigma, for example from their supervisor or peers, can pose additional hurdles to a student in need of assistance (Dunn et al., 2008). Access to online resources offers anonymity, which can bypass those barriers to accessing support.

When a student expresses concerns about their own mental health, loved ones and colleagues alike can benefit from being able to quickly access the university’s resources online. If the student has moved away to university, guidance from their supervisor and peers can be especially important and feeling supported by a mentor has been cited as a crucial indicator for a graduate student completing their training (Sowell et al., 2008). Resources that are accessible online can help advisers to learn about current issues and provide informal mental health support for their trainees without necessarily taking additional training.

Online platforms have the power to connect people to form communities even if they are separated by geography. By providing a community where everyone can share their own experiences, it helps others know they are not alone in their experiences. This sense of community and shared experience can assist in decreasing stress and lowering the sense of "hopelessness" in an individual’s own struggle. PhD Balance provides this community through sharing member-submitted and curated stories. Additionally, it gives a space for people to discuss their own struggles and crowd-source advice through several social media outlets.

With an estimated one mental health specialist for every 1,700 students, front-line support – like student services offices – is often created to serve the "average" student demographic (Winerman, 2017); however, members of different populations require different interventions, which could be supplemented through providing online resources. Minorities often face additional challenges related to a feeling of "otherness" and a lack of access to resources (Hunt and Eisenberg, 2010). Underrepresented minority (URM) students are less likely to have adequate mentorship and personal support, potentially limiting their success (Sowell et al., 2015), and international students are less likely to use mental health services (Hyun et al., 2007). Web pages can be translated into various languages to allow students and their families to gain access to resources even if no one in the human support services is fluent in their primary language. Lastly, people with disabilities can also benefit from the use of online resources that are optimized for accessibility; students with mobility issues do not have to visit an in-person facility, screen-readers provide information to those with vision impairment, and web pages can be easily read by people with a hearing impairment.

Many online resources created and optimized by larger institutions could likely be amended to fit the needs of a new university more quickly than changing in-person systems. Rather than recreating the wheel, universities might be able to model their own sites on others or even share tools and strategies (Box 1). Updates to reflect changes in needs, policies or best practices can also be made immediately for online resources. One institution that can be used as a model for easy access to mental health services and online resources is the University of Michigan and its associated Rackham Graduate School.

Box 1. A first glance at online resources.

Initial online searches (within the United States) for mental health resources will route you to the sites for the National Institute for Mental Health, the Substance Abuse and Mental Health Services Administration, National Alliance on Mental Illness, and other similar national organization websites. These sites direct users to 24-hour helplines, crisis resources and resources to find health-care providers. Another search hit lists "80 Awesome Mental Health Resources When You Can’t Afford a Therapist" and includes online forums, places to meet support groups, and apps that can help with guided meditation, online therapy sessions and self-guided behavioral therapy techniques (Schreiber, 2015). Other online support systems tailored to graduate students, such as Beyond the Professoriate and PhD Balance, can help students succeed both in terms of personal and professional growth, and there are many blogs focused on these topics too. Identifying and providing access to these types of resources can supplement university-specific initiatives and guide future resource creation.

## Limitations of online resources

While we think there is a strong case for universities using online tools to help the mental wellbeing of their graduate students, inappropriate use or implementation of such resources can result in negative consequences. Institutions need to remain aware of their limitations. Firstly, these measures should never be used as a replacement for other types of existing services. Online resources may provide users with a false sense of resolution, and psychoanalyst and psychiatrist Mary Davis warns that reliance solely upon online interventions, including "meeting" with a teletherapist, may not be sufficient to address mental health issues if the patient does not open up as readily in an online setting (Weiss, 2018). Instead, rather than considering online services to be a solution, they may be better seen as temporary stopgaps and pathways to other measures that will see people receiving appropriate support.

Established online resources must also be maintained to ensure that are easy to navigate and that students do not face sudden barriers to care due to broken or missing links. Further, separate departments and colleges within a university system must coordinate to keep up with changes in resources between web pages, which may require universities to dedicate money and time to maintain their digital resources.

Although most of us are reading this article via the web, internet access must not be taken for granted. About 10% of Americans do not use the internet (Perrin and Kumar, 2019), and while graduate students do not necessarily reflect the overall population, many in their support networks still rely on phone or in-person routes to get resources. To address this, resources created on the internet can be modified to suit print dissemination (and vice-versa) to enable students of all backgrounds to access support.

## Recommendations

We would make the following recommendations for universities looking to support the mental wellbeing of their graduate students through the curation of online resources. First, all resources should originate from one centralized location, allowing students to find them with minimal 'clicks' from the same starting location instead of using general search engines. Second, university systems already in place for undergraduate and professional school students (like medical students) could guide the organization and content of those tailored for graduate students, including the ways in which they are often sponsored through university funds. For instance, graduate schools could take inspiration from the Wellbeing Index for Physicians, which was created by the Mayo Clinic and sanctioned by the American Medical Association and allows doctors and medical students to evaluate their mental health over time and find local and national resources applicable to their current situation. Third, online resources should be accompanied by contact information with appropriate human resources so the user can access further assistance. Finally, institutions can gauge the efficacy of online support initiatives through collection of metrics, both to improve the systems and to provide evidence for their continued support. Some metrics to be considered are the number of students who access the online resources; whether these website interactions lead students to contact appropriate support offices; and if students report awareness of and satisfaction with the online content.

## Conclusion

We have seen firsthand how online resources can help graduate students struggling with their mental health. We would like to see more institutions deploy them as part of their wider provisions to support mental wellbeing of their different student populations. We hope that their potential to remove the barriers that may limit current access to appropriate support will mean that no graduate student is left struggling without help.

## Note

This Feature Article is part of a collection on Mental Health in Academia. The collection also includes a survey into the experiences of people who have helped researchers who are struggling with their mental health.
